# One-step wet chemical synthesis of gold nanoplates on solid substrate using poly-l-lysine as a reducing agent

**DOI:** 10.1016/j.mex.2018.12.002

**Published:** 2018-12-04

**Authors:** Suratun Nafisah, Marlia Morsin, Nur Anida Jumadi, Nafarizal Nayan, Nur Zehan An’nisa Md Shah, Nur Liyana Razali, Muhammad Mat Salleh

**Affiliations:** a Microelectronics & Nanotechnology - Shamsuddin Research Centre (MiNT-SRC), Institute of Integrated Engineering, Universiti Tun Hussein Onn Malaysia, 86400, Parit Raja, Batu Pahat Johor, Malaysia; b Department of Electronics, Faculty of Electrical and Electronic Engineering, Universiti Tun Hussein Onn Malaysia, 86400, Parit Raja, Batu Pahat Johor, Malaysia; c Institute of Microengineering and Nanoelectronics (IMEN), Universiti Kebangsaan Malaysia, 43600, Bangi, Selangor, Malaysia

**Keywords:** One-step wet chemical synthesis of Gold Nanoplates on Solid Substrate Using Poly-l-lysine as Reducing Agent, Localized surface plasmon resonance, Gold nanoparticles, Gold nanoplates, Plasmonic sensor

## Abstract

A one-step wet chemical approach or seedless growth process has several advantages compared to the traditional seed-mediated growth method (SMGM), such as being simpler and not requiring a multistep growth of seeds. This study had introduced a one-step wet chemical method to synthesis gold nanoplates on a solid substrate. The synthesis was carried out by simply immersing clean ITO substrate into a solution, which was made from mixing of gold chloride (precursor), cetyltrimethylammonium bromide or CTAB (stabilizing agent), and poly-l-lysine or PLL (reducing agent). Consequently, the size of the nanoplates in the range of (0.40 – 0.89) μm and a surface density within the range (21.89–57.19) % can be easily controlled by changing the concentration of PLL from 0.050 to 0.100 w/v % in H_2_O. At low PLL concentrations, the reduction of the gold precursor by PLL is limited, leading to the formation of gold nanoplates with a smaller size and surface density. The study on the sample by using energy-dispersive x-ray spectroscopy (EDS) confirmed that gold peaks occurred. The optical properties of the samples were examined by a UV–vis Spectrophotometer and showed that there was no strong surface plasmon resonance band observed at UV–vis and infrared regions, which agreed to micron-sized gold nanoplates.

• Gold nanoplates synthesized on the substrate using a simple one-step wet chemical synthesis approach with poly-l-lysine (PLL) as a reducing agent and CTAB as a stabilizing agent.
• The nanoplate’s size and surface density was strongly dependent on the concentration of PLL.
• Gold nanoplates synthesized using PLL with a concentration 0.050% showed perfect triangular shape, less by-products and more homogenous in size.

## Graphical abstract

**Figure:**
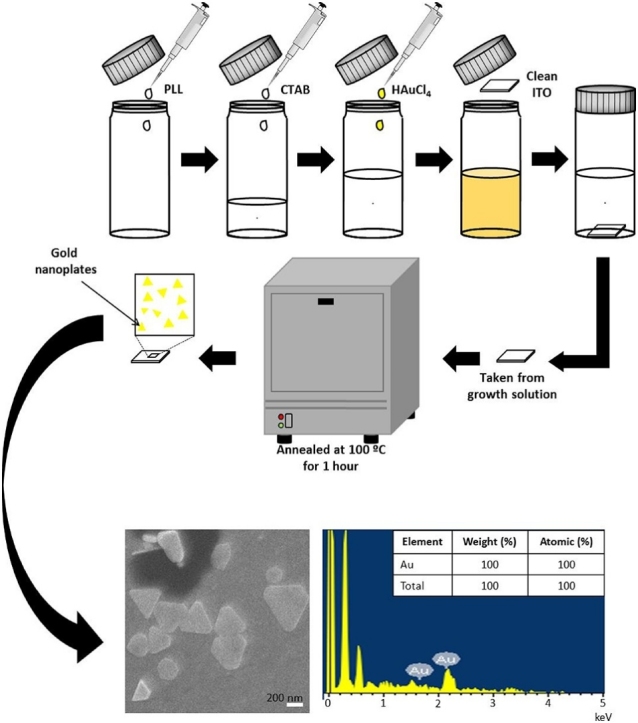

**Specifications Table**

**Table:** 

Subject area	Materials Science
More specific subject area	*Plasmonic*
Method name	*One-step wet chemical synthesis of Gold Nanoplates on Solid Substrate Using Poly-*l*-lysine as Reducing Agent*
Name and reference of original method	*Seed-mediated growth method (SMGM)*

## Method details

A previous work by the author of this study reported that the synthesization of gold nanoplates had formed by using the SMGM [[1], [2], [3], [4]]. In this method, the pre-treatment of the sample was carried out by immersing the ITO substrate into the PLL solution prior to the seed and growth process. Despite the fact that critical parameters and randomness of crystal growth can be easily controlled in SMGM [5], it still requires multistep processes and thus, ineffective in terms of time and materials. These disadvantages are overcome by using the seedless or one-step method. Some researchers have reported a new strategy for synthesizing gold nanoplates using one-step method. For example, Huang et al. developed a method for synthesizing gold nanoplates using the one-step approach via thermal reduction of gold ions with trisodium citrate in the presence of CTAB. In their study, various sizes of gold nanoplates were tuned by varying growth parameters, such as temperature, reaction time, and reagent concentration [6]. L. Chen et al. reported that the one-pot seedless process for the synthesis of gold nanoplates that employedoxidative etching method using iodide ions played a role as both etching and capping agents [7]. Alternatively, S. Chen et al. proposed a one-step approach for rapid synthesis of gold nanoplates by reducing gold ions in the presence of CTAB. The difference with Huang et al. was that they used ascorbic acid instead of trisodium citrate as a reducing agent [8]. Recently, Kutnerr et al. proposed the seedless technique to synthesis gold nanoplates in triangular shape using 3-butenoic acid (3BA) and benzyldimethylammonium chloride (BDAC) as reducing and capping agents, respectively [9]. However, all these techniques only successfully grew gold nanoplates in aqueous solution form. Thus, for a wider implementation in some applications, such as sensor and non-linear optics, it is still necessary to grow gold nanoplates on a solid substrate. As far as this study is concerned, no study has ever reported on synthesis gold nanoplates on a solid substrate using the one-step method. Herein, this study proposed the one-step method to produce gold nanoplates on solid substrate i.e. ITO by introducing PLL as a reducing agent and CTAB as a stabilizing agent during growth process. Additionally, this technique does not require the seed process as well as complicated procedures or equipment. Hence, by using this technique, the size of nanoplates can be easily controlled by changing the concentration of PLL. The materials used were Poly-l-lysine (PLL, 0.100 w/v % in H_2_O), cetyltrimethylammonium bromide (CTAB, ≥ 98%) and hydrogen tetrachloroaurate (HAuCl_4_, ≥ 99.9%). All these materials were purchased from Sigma Aldrich, USA. The solutions of these chemicals were prepared using de-ionized water (DIW) with a resistivity of 18.2 MΩcm. Prior to use, all glassware were washed with soap and water, sonicated in DIW, acetone and 2-propanol for 15 min respectively, and dried.

### Substrate preparation

**Fig. 1:**
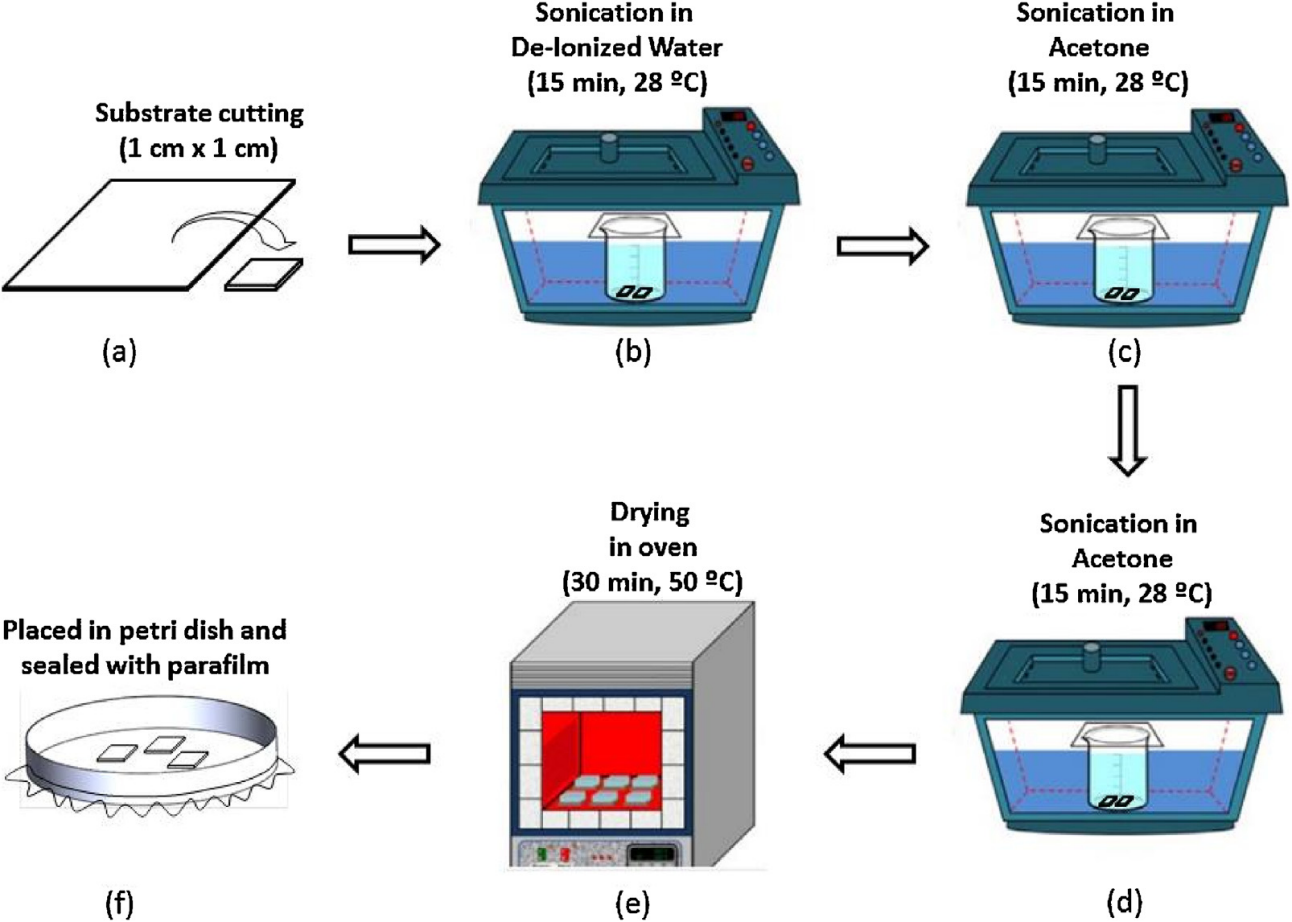
Schematic of substrate preparation.

Substrate preparation was done to ensure it was cleaned from any contaminants before it could be used in subsequent processes. The substrate used in this study was indium tin oxide (ITO). The schematic of the substrate preparation is shown in Fig. 1. The initial preparation involved the cutting of the ITO into a desire size, i.e. 1 cm × 1 cm (Fig. 1 process a). Next, the ITO was immersed in DIW and sonicated for 15 min (Fig. 1 process b). This process was repeated for acetone and 2-propanol, respectively (Fig. 1 process c and d). Then, the ITO was taken out from the solution and dried in an oven with a temperature 50 °C for 30 min (Fig. 1 process e). Lastly, the substrate was placed in a petri dish and sealed with parafilm before being used in the next process (Fig. 1 process f).

### Synthesis of gold nanoplates

**Fig. 2:**
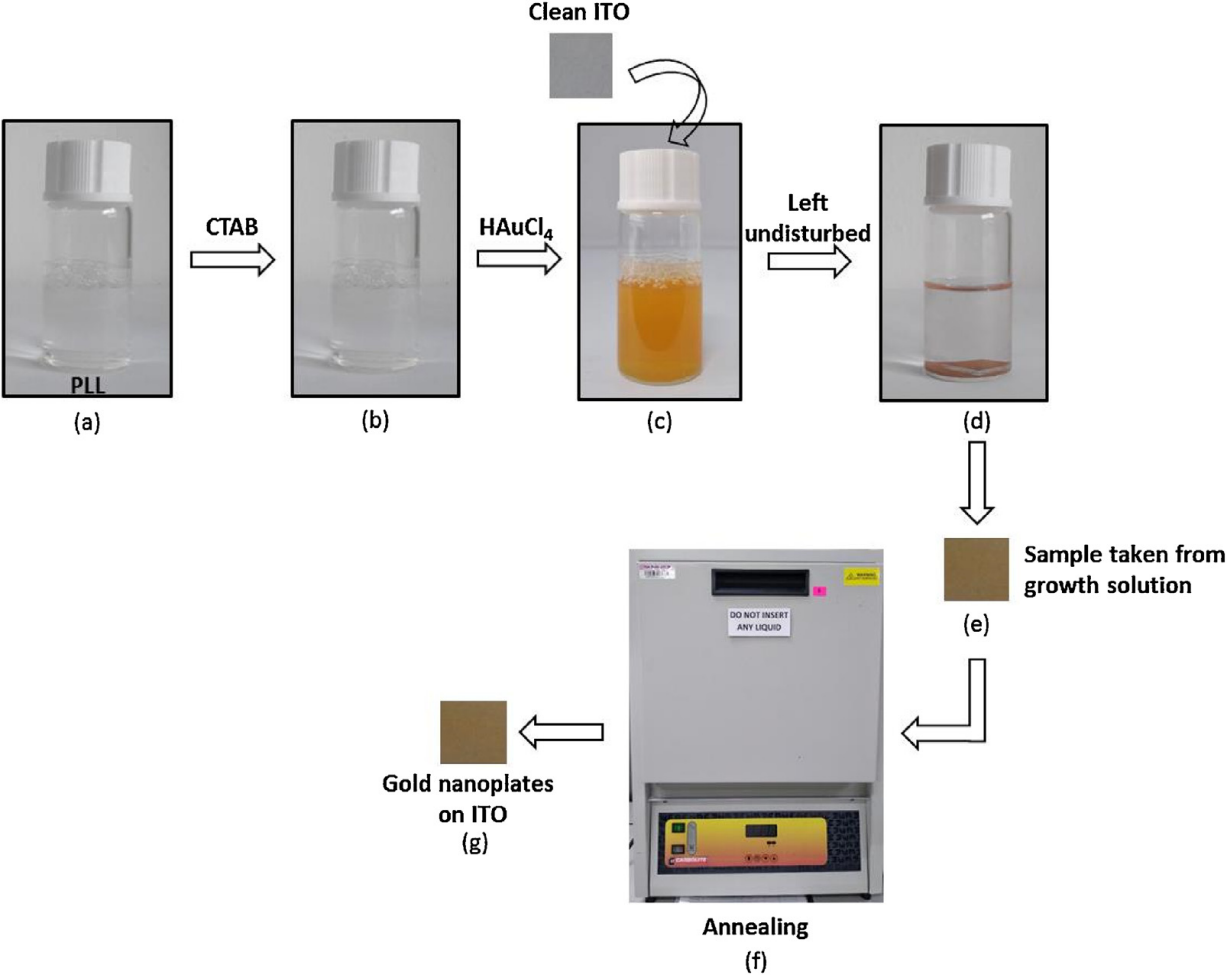
Synthesis of gold nanoplates on ITO substrate using one-step wet chemical method.

Gold nanoplates were synthesized by immersing clean ITO substrate in the aqueous solution containing 2.5 ml PLL, 2.5 ml CTAB 0.1 M, and 1 ml HAuCl_4_ 0.01 M (Fig. 2 process a to d) at room temperature for 15 h. After that, the sample was rinsed in DIW several times and dried (Fig. 2 process e). Finally, the sample was annealed at 100 °C for 1 h in order to remove surfactant and organic compounds (Fig. 2 process f).

This research hypothesized that the formation of gold nanoplates and their sizes are strongly dependent on PLL concentration. In order to study the effect of PLL concentrations, three different samples corresponding to different concentrations were prepared, i.e. 0.050, 0.075, and 0.100%. The PLL sample at 0.100% concentration was used as received without further dilution. Meanwhile, the PLL with concentrations of 0.075% and 0.050% were prepared by diluting 1.875 and 1.250 of PLL stock solutions (PLL with concentration 0.1%) with a solvent comprising H_2_O (DIW) to bring the final volume up to 2.5 ml, respectively.

The surface morphology of the samples was obtained using a Jeol JSM-7600 F Schottky FESEM, USA with an accelerating voltage of 5 kV. Elements of the samples were investigated using the energy-dispersive X-ray spectrometer (EDS) detector in the FESEM. UV–vis absorption spectra were recorded by using the UV-1800 Shimadzu UV–vis Spectrophotometer from Japan at a wavelength range of 300 to 1000 nm.

Hence, when the AuCl^−^ and CTAB were mixed without adding the PLL solution, the colour of the mixture will turned from light yellow to orange indicating the formation of only AuBr^−^. Moreover, the colour of this mixing solution remains unchanged when left for a long time. Thus, no formation of gold nanoparticles were traced in the solution [10]. On the other hand, when PLL, which has positive charge, was presented in this mixture, it was spontaneously absorbed on to the AuCl- and formed a mixed layer [11]. In this condition, PLL plays a role as a reducing agent, which reduces Au(3+) to Au(0), as observed when the solution slowly changed from orange to colorless [12] (Fig. 2 process c to d). Furthermore, Au(0) acts as a supply material for nucleation and then grows favorably in a specific direction and forms gold nanoplates on the substrate and at the bottom of the vial (Fig. 1d). During the growth process, the colour of sample slowly changed from colourless to yellow mustard (Fig. 1e), which indicated the formation of a gold film [13].

**Fig. 3:**
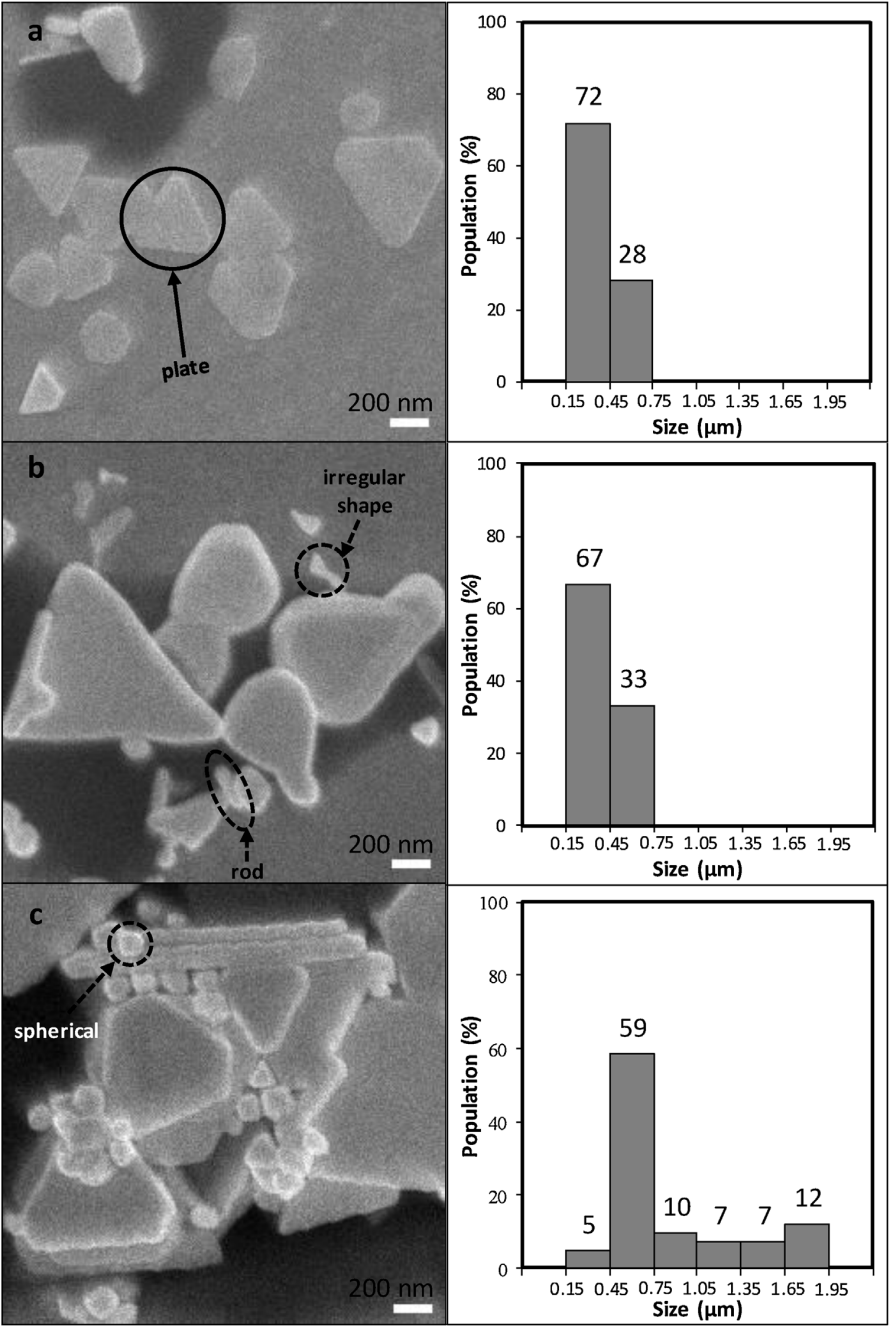
FESEM images (left) and corresponding size distribution diagram (right) of gold nanoplates synthesized and obtained at PLL concentrations of a) 0.050, b) 0.075, and c) 0.100%. The highlighted nanoparticles by solid and broken circles respectively represent nanoplates and by-products.

In this proposed method, the sample was immersed in the reaction solution for 15 h at low, intermediate, and high PLL concentrations, i.e. 0.050, 0.075, and 0.100%, respectively. FESEM images of the gold nanoplates synthesized at various PLL concentrations are shown in Fig. 3. From these images, the analysis for shape, size and surface density of nanoplates and by-products were carried out using ImageJ. Fig. 3 shows that gold nanoplates were successfully grown in mainly shapes were triangular in all PLL concentrations. Besides that, by-products in various shapes were also formed on the sample, such as spherical, rod, and irregular shapes.

As shown in Fig. 3, the nanoplates were mostly triangular in structure. Based on the analysis, the average size of gold nanoplates were 0.40 ± 0.01 μm, 0.71 ± 0.04 μm, and 0.89 ± 0.07 μm for the sample prepared at PLL concentrations of 0.050, 0.075, and 0.100%, respectively, as listed in Table 1. This result indicates that not only the gold nanoplates were successfully formed but also the size of nanoplates was strongly dependent on the PLL’s concentration. Higher concentrations of PLL leads to a bigger size of gold nanoplates.

**Table 1:** Average size and surface density of all samples.

No.	Concentration of PLL w/v (%)	Size (μm)	Surface density		Ratio of surface density nanoplates and byproduct
			Nanoplates (%)	Byproduct (%)	
1.	0.050	0.40 ± 0.01	21.89 ± 0.97	0.65 ± 0.14	33.68 ± 0.26
2.	0.075	0.71 ± 0.04	31.04 ± 3.66	1.69 ± 0.17	18.37 ± 0.22
3.	0.100	0.89 ± 0.07	57.19 ± 5.59	6.56 ± 1.53	8.72 ± 0.33

This study also found that lower concentrations of PLL (0.050%) led to more perfect triangular shaped gold nanoplates (see Fig. 3a). However, the PLL concentration was not always favourable to maintain the perfect triangular shape of gold nanoplates. When the concentration of PLL increased to 0.075%, the triangular shape changed or became imperfect (see Fig. 3b). Then, when the PLL concentration continuously increased to 0.100%, the nanoplates, although perfectly triangular, were inhomogeneous in size with excessive by-products (see Fig. 3c). The relative amount of PLL had a significant effect on the growing shape of nanoplates. The reason probably that at a lower concentration of PLL (0.050%), the formation rate of Au(0) is as slow as the nucleation rate, meanwhile at high concentrations of PLL (0.100%), the reduction rate of Au(3+) to Au(0) was as fast as the nucleation process. Both of these conditions resulted in a perfect triangular shape. However, the condition was quite different at a medium concentration of PLL (0.075%), in which the velocity of gold nanoplate nucleation rate was lower than the Au(0) reduction rate. In other words, there were more Au(0) per nucleation and thus, provided additional shape to the gold nanoplates. This condition resulted in nanoplates with an imperfect triangular shape.

Then, surface density of gold nanoplates and by-products was independently determined by measuring the area covered by the growth divided by total surface area. The calculations were carried out using ImageJ. Three different areas, which are 1302.49 μm^2^ respectively, were measured and analysed to determine the average surface density of nanoplates and by-products. The results are shown in Table 1. The highest surface density of the nanoplates was obtained from the sample prepared with a PLL concentration of 0.100%. As the PLL concentration decreased, the density of the nanoplates also decreased. This behaviour is assumed based on the fact that at high PLL concentrations, the reduction process of the gold precursor is faster compared to lower PLL concentrations and thus, resulting in more formation about the gold nuclei at initial growth condition. It is also interesting to note that under higher PLL concentrations, higher by-products are obtained. It is presumed that at higher PLL concentrations, large amounts of gold precursors are reduced by PLL during the reaction. At such high reduced-gold, there are plenty of gold resources available for the formation of nanoparticles in many different structures. The ratio of nanoplates to by-products was calculated since the surface density of nanoplates and by-products increase with the increase in PLL concentrations. The results show that the lowest concentration of PLL provides the highest ratio of nanoplates to the by-products, which indicates that the lowest PLL concentration is the best condition to grow nanoplates. In order to minimize the quantity of by-products obtained, other synthesis parameters such as growth time can be controlled as investigated in previous studies [1,4].

**Fig. 4:**
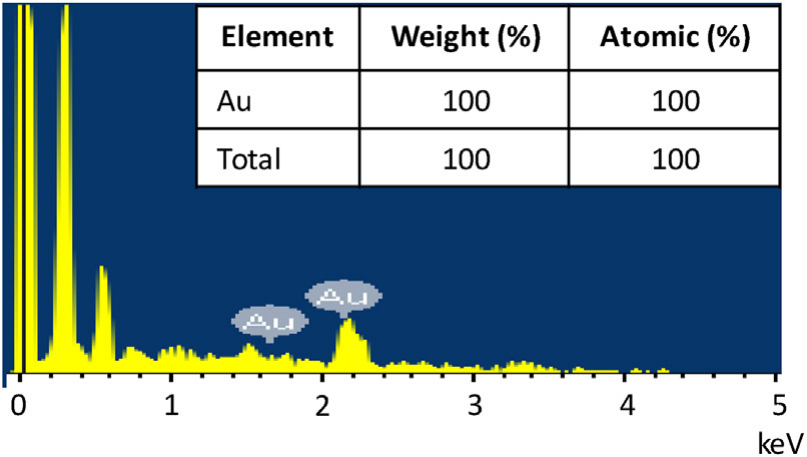
Energy-dispersive x-ray spectroscopy of a gold nanoplates from the sample synthesized at 0.050% PLL concentrations.

In addition, the elemental composition of nanoplates prepared at a PLL concentration of 0.050% was further characterized by energy-dispersive X-ray Spectroscopy (EDS) and the result is shown in Fig. 4. The presence of gold elements, as shown in the EDS spectrum with the weight had 100% confirmed the formation of gold nanoplates.

**Fig. 5:**
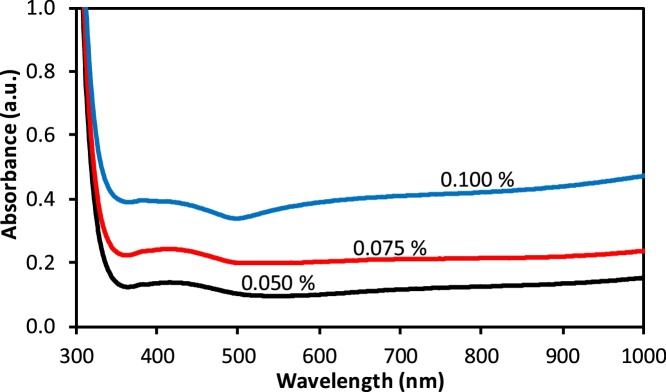
UV–vis absorption spectra of the sample synthesized at three different PLL concentrations.

Optical properties of the nanoplates are characterized by UV–vis absorption spectroscopy. Fig. 5 shows the UV–vis absorption spectra of the three samples synthesized at different PLL concentrations. The absorption spectrum of all gold nanoplate samples showed a steep increase in absorption of above 500 nm. However, steady increments were observed for samples prepared at PLL concentrations of 0.050 and 0.075%. The absorption spectrum also showed that there was no strong surface plasmon resonance band observed at the UV–vis and infrared regions due to the micron size of gold nanoplates, which provides the corresponding characteristic of UV–vis spectra as a bulk size. This result is consistent with previous spectrum observations for micron-sized gold nanoplates [8]. Conversely, narrow and sharp peaks of gold nanoplates’ UV–vis spectra could be obtained if nanoparticles were nanometer in size. Besides that, the broad absorption band of gold nanoplates could also be ascribed to the non-uniform shape and size of nanoplates.
