# Supplementary material for: One-step wet chemical synthesis of gold nanoplates on solid substrate using poly-l-lysine as a reducing agent
Source: MethodsX. 2018 Dec 4;5:1618–25. doi: 10.1016/j.mex.2018.12.002 (PMC6290129; doi:10.1016/j.mex.2018.12.002)
Supplement: Supplementary file 1 [file mmc1.docx]

**Additional information**

The thickness of nanoplates was around 25.57 nm, obtained through line profile calculation of the AFM image, as shown in Fig. 5. It is assumed that the thickness of gold nanoplates have no significant difference between samples prepared at PLL concentration of 0.050, 0.075, and 0.100 %.


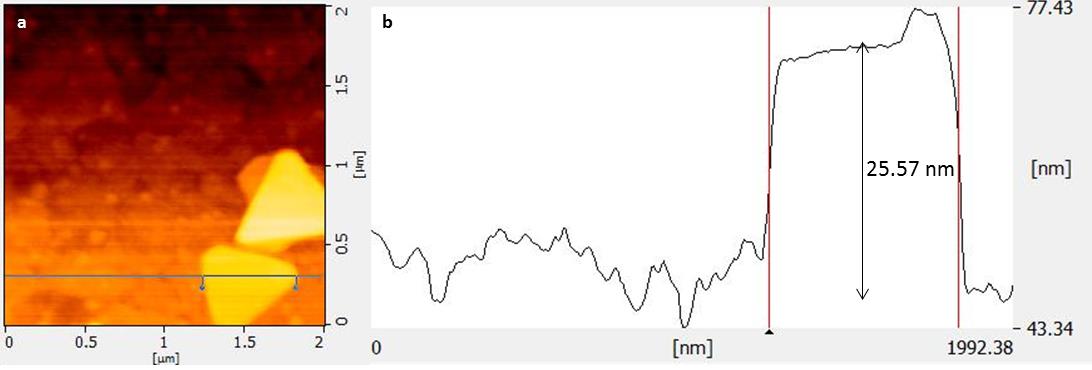


Fig. 6. (a) AFM image of gold nanoplates and (b) its line profile.
